# Transthyretin and complex protein pattern in aqueous humor of patients with primary open-angle glaucoma

**Published:** 2008-08-04

**Authors:** F. H. Grus, S. C. Joachim, S. Sandmann, U. Thiel, K. Bruns, K. J. Lackner, N. Pfeiffer

**Affiliations:** 1 Experimental Ophthalmology, Department of Ophthalmology, Johannes Gutenberg University, Mainz, Germany; 2 Department of Clinical Chemistry and Laboratory Medicine, Johannes Gutenberg-University, Mainz, Germany

## Abstract

**Purpose:**

To analyze protein patterns in the aqueous humor of glaucoma patients in comparison to control subject using two different methods.

**Methods:**

Aqueous humor was collected from 52 patients with primary open-angle glaucoma (POAG) and from 55 control subjects (CO). Twenty-two POAG samples and 24 CO samples were used for protein profiling through surface enhanced laser desorption/ionization-time of flight-mass spectrometry (SELDI-TOF-MS) ProteinChip arrays. The data were analyzed by multivariate statistical methods and artificial neural networks. One highly significant biomarker was identified through matrix assisted laser desorption/ionisation time of flight-mass spectrometry (MALDI-TOF). Thirty samples from patients with POAG and 31 control samples were analyzed through two-dimensional electrophoresis. Subsequently, the protein spots of all gels were detected, and the two groups were compared. One spot group exhibiting clear differential abundance was identified by mass spectrometry (electrospray ionization mass spectrometry).

**Results:**

In the samples analyzed by SELDI-TOF-MS, about 250 protein peaks could be consistently clustered in both groups. The analyses revealed eight biomarkers, which discriminated glaucoma from non-glaucoma controls with a sensitivity of 90% and a specificity of 87%. These biomarkers were purified further, and one marker, which was upregulated in glaucoma patients (p=0.006), was identified as transthyretin. The upregulation of transthyretin in POAG patients was also confirmed by enzyme linked immunosorbent assay (ELISA; p=0.03). In all samples analyzed by two-dimensional electrophoresis, complex protein patterns were detected in a total of 177 spot groups. The aqueous humor of all glaucoma patients revealed some regions that were clearly different from the controls. Several spots were significantly increased in the aqueous humor of glaucoma patients. One of the proteins that is highly abundant in the aqueous of glaucoma patients was identified as transthyretin.

**Conclusions:**

The aqueous humor of glaucoma patients revealed characteristic differences in protein/peptide profiles from control patients using two different analytical methods, SELDI-TOF-MS and two-dimensional electrophoresis. Interestingly, we could detect elevated transthyretin concentrations in glaucoma samples. Transthyretin might play a role in the onset of glaucoma since it has been shown to form amyloid deposits. These particles could cause outflow obstructions thereby increasing intraocular pressure as a possible onset mechanism.

## Introduction

Glaucoma is one of the major causes of visual impairment and blindness worldwide [1]. It describes a heterogeneous group of eye disorders that are usually characterized by typical structural and functional abnormalities of the optic disc, retinal nerve fiber layer, and visual field. The pathogenesis and mechanisms of glaucoma are still not fully explained. Several possible mechanisms are discussed that include a mechanical pressure component, vascular dysregulation [2,3], oxidative stress [4], or an autoimmune component [5]. One of these mechanisms could be traced through abnormalities in the protein composition of eye tissue and aqueous humor [6-8].

Aqueous humor, the fluid in the anterior and posterior chamber of the eye, plays an important role in maintaining functions of the eye like refraction, shape, and intraocular pressure (IOP) [9]. A block of the aqueous humor outflow can cause an increase in IOP [10], which is the major risk factor for glaucoma [11]. Since the eye is one of the immune privileged regions of the body [12], it might be very interesting to analyze the liquid that is closer to the site of glaucoma damage than serum. Some proteins that are upregulated in glaucoma patients are already identified, like metalloproteinase [13,14].

One of the challenges of protein analysis in aqueous humor samples is the limited amount of samples that can be obtained from one patient. One can collect about 100–150 µl of aqueous from one subject [15,16]. Another difficulty arises from the low protein concentration, which is approximately 0.20–0.5 mg/ml [17]. Therefore, very sensitive methods such as two-dimensional (2D) gel electrophoresis and surface enhanced laser desorption/ionization-time of flight-mass spectrometry (SELDI-TOF-MS) need to be used for protein analysis in aqueous. 2D gel electrophoresis has already been used to analyze aqueous humor samples from patients with acute corneal rejection [15] or myopia [18].

The aim of this study was to analyze the complex protein patterns in the aqueous humor of patients with primary open-angle glaucoma. We used SELDI-TOF-MS and 2D gel electrophoresis for protein separation and have identified some important proteins through mass spectrometry.

## Methods

### Patient classification

All patients included in this study were undergoing cataract or glaucoma surgery (e.g., trabeculectomy) and had complete ophthalmologic examinations at the Department of Ophthalmology at University of Mainz (Mainz, Germany). The patient classification was done in accordance with the guidelines of the European Glaucoma Society [11]. The control patients (CO, n=55) had no other ocular disorders besides cataract. They had no history of glaucoma, no pathologic fundus, and no elevated IOP.

The diagnostic criteria for primary open-angle glaucoma (POAG; n=52) were the presence of glaucomatous optic disc damage with corresponding glaucomatous changes in the visual field (examined by Goldmann perimeter; Haag-Streit, Wedel, Germany), the presence of open angles in the patient’s eyes, and the absence of alternative causes of optic neuropathy (e.g., infection, inflammation, meningeal disease, ischemic disease, and compressive lesions). All POAG patients had an intraocular pressure greater than 21 mmHg without treatment (Goldmann applanation tonometer; Haag-Streit). The IOP was calculated from three separate measurements. To rule out wrong IOP measurements, a pachymetry examination was also done.

### Sample collection

All aqueous humor samples (AH) were collected at the beginning of the surgery. After the corneal incision, the aqueous humor fluid was removed from the anterior chamber under sterile conditions by using a tuberculin syringe, and 50-100 µl per sample was collected. All surgical procedures were performed by experienced ophthalmic surgeons. After removal, the AH was stored in microtubes at −80 °C until analysis. Before sample collection, written consent was obtained from every patient. This study was approved by the local ethics committee and followed the principles of the Declaration of Helsinki.

### SELDI-TOF-MS analysis

Twenty-two aqueous humor samples from POAG patients (mean age 66.4±14 years) and 24 control samples (mean age 70.5±13 years) were measured with SELDI-TOF-MS. All samples used for the SELDI-TOF-MS analysis were applied to two different chromatographic surfaces, a weak cation exchange surface (CM10) and a reversed phase chemistry hydrophobic chip (H50). AH (2 μl) was applied on each spot and air dried. Then, 1 µl of a saturated sinapinic acid in 50% acetonitrile/0.1% trifluoroacetic acid was applied to each spot twice. After air drying, each spot was processed in the ProteinChip Reader.

ProteinChip arrays were analyzed on a PBS-IIC ProteinChip Reader equipped with an array autoloader using the ProteinChip Software version 3.2 (all from Bio-Rad, Hercules, CA). Each array was read at two laser intensities, low intensity to optimize for lower molecular weight proteins and high intensity for higher molecular weight proteins. The high intensity protocol used an average of 192 laser shots to each spot with a laser intensity of 220 (in relative units), a deflector setup of 2,000 Da, and a molecular weight detection range of 3,000-200,000 Da. The focus mass was set at 20,000 Da. The detector was run at a sensitivity of nine. For low intensity measurements, the laser intensity was set to 200 (in relative units) and a deflector limit of 1,500 Da, and the focus mass was set at 8,000 Da. The raw data was transferred to CiphergenExpress 2.1 database software (Bio-Rad, Hercules, CA) for workup and analysis. The baseline was subtracted using a setting of 12 times the expected fitting width. Spectra intensity was normalized using the total ion current against an external normalization coefficient according to the manufacturer.

The total ion current includes the total area under the curve including both peak area and inherent noise. Intensity normalization was done by calculating the total ion current for a spectrum, and the total ion current was then divided by the number of data points for that spectrum to obtain an average ion current for each individual spectrum. An external coefficient of 0.2 was chosen against which each spectrum was normalized. The very low mass region contains chemical “noise” from the matrix and was therefore excluded from the analysis. The low mass cutoffs were 3,000 m/z for low laser intensity measurements and 8,000 m/z for high laser intensity measurements.

Automatic peak detection was performed using five times the signal-to-noise ratio for the first pass of the peak detection and two times the signal-to-noise ratio for the second pass. The first pass uses low sensitivity to detect obvious and well defined peaks. The second pass uses higher sensitivity settings to search for smaller peaks with the mass values found in the first pass.

A list of peak cluster was created from these detected peaks. A peak cluster was created if a peak was found in 20% of all spectra for an individual condition above the first pass cutoff. The mass window for peak clustering was defined as 2% of the peak mass for spectra read at high laser intensity and 0.3% of the peak mass for spectra read at low laser intensity.

### 2D gel electrophoresis

Aqueous humor samples (30) from POAG patients (mean age 72.6±10 years) and 31 control samples (mean age 71.8±10 years) were separated by 2D gel electrophoresis as described previously for tears [19-21]. Thirty microliters of AH were used for each separation since this concentration resulted in the highest resolution with about 225 spots between a 10-80 kDa range and pH 4–7, which was tested before this study.

AH was mixed with 100 µl of rehydration buffer containing 8 M urea, 2% CHAPS (Cholamidopropyldimethylamoniopropanesulfate), 0.28% DTT (Dithiothreitol), 0,5% immobilized pH gradient (IPG) buffer (pH 4-7 linear), and 0.002% bromophenol blue. This solution was used to rehydrate IPG strips with linear pH 4–7 (Immobiline Dry-strips; Amersham Biosciences, Munich, Germany) in ceramic strip holders for 14 h. Isoelectric focusing (first dimension) was performed on an Ettan IPGphor (Amersham Biosciences, Munich, Germany).

After rehydration, the IPG strips were loaded with 130 µl sample solution and electro-focused on experimentally determined running conditions. Voltage increased in gradient and in step’n’hold steps from 200 V to 8,000 V over a 9.5 h period.

After finishing the IEF, the focused strips were incubated in an equilibration buffer (50 mM Tris hydrochloride, 6 M urea, 30% glycerol, 2% sodium dodecyl sulfate, 0.002% bromphenyl blue, and 1% DTT) for the second dimension (SDS–PAGE). The vertical SDS–PAGE was performed using 13.5% polyacrylamide gels (duration: 60 min). Molecular mass marker proteins with a range of 10–120 kDa (Broad Range; Bio-Rad, Munich, Germany) were run together with each sample. The gels were visualized using a silver staining protocol according to Oakley [22]. Gels were fixed in 50% methanol and 5% acetic acid, and they were then washed two times in 50% methanol and pure water to reduce background staining. Afterwards they were shortly sensitized in 0.02% sodium thiosulfate pentahydrate and subsequently washed two times before the staining reaction was started with 0.1% silver nitrate. Finally, the gels were washed in water and developed in 0.04% formalin. The staining reaction was stopped in 5% acetic acid. A BioDocAnalyze system (Biometra, Goettingen, Germany) was used to generate digital images of the stained gels.

### Data analysis

#### SELDI-TOF measurements

The cluster lists containing peak intensity values for each group were exported as ASCII files to Statistica (version 7.1; StatSoft, Tulsa, OK). Based on these normalized peak intensities, p values based on *t*-tests and multivariate analysis of discriminance were calculated.

The Statistica software performed a multivariate analysis of discriminance based on these peak clusters and different conditions (surface type and laser energy). This analysis of discriminance selected eight important protein biomarkers to discriminate between both groups.

An artificial neural network (ANN) with the panel of these biomarkers was created. Artificial neural networks learn through experience and not through programming; their construction is comparable to that of the human brain [23]. One of the abilities of neural networks is to accurately predict data that were not part of the training data set, a process known as generalization. At first, a set of protein profiles was used to train the network. Unknown spectra is then placed in the glaucoma or non-glaucoma groups because of the criteria learned from the other spectra. This network is typically formed by three layers. The input layer receives information from outside via input sources. The output layer communicates the results via dedicated output devices. A layer of hidden units lies between them and does most of the processing. ANNs can be used to distinguish between pathogenetic and nonpathogenetic proteins [24].

The software generates a receiver operating characteristics (ROC) curve, a graphical description between sensitivity and specificity. The x-axis shows 1–specificity and the y-axis the sensitivity. The area under the curve (AUC) is the quality factor for this test. An ideal AUC would have an r value of 1. If the AUC r value is below 0.5, the classification is only done by coincidence.

#### 2D  gels

**Figure 1:**
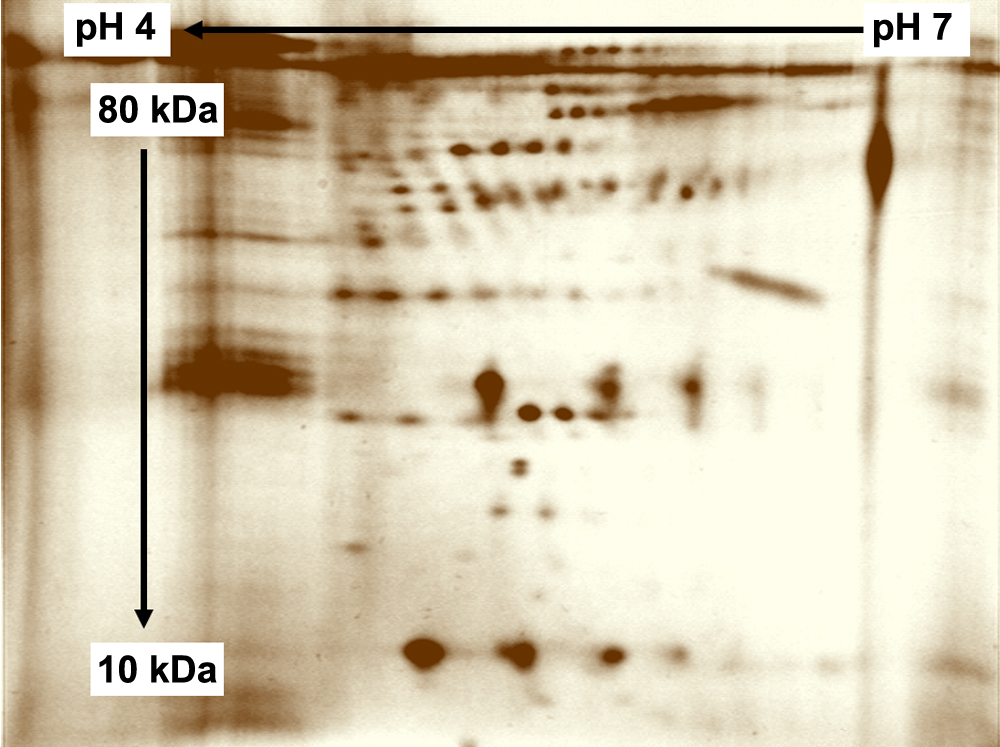
Example of a two-dimensional gel with Oakley silver staining of aqueous humor sample from a POAG patient. The x-axis shows the first dimension (separation by isoelectric point), and the y-axis shows the second dimension (separation by molecular weight). The dark spots represent stained proteins.

Digital images of the 2D gels were analyzed using ImageMaster^TM^ 2D Platinum Software (Amersham Biosciences, Munich, Germany). A 2D gel standard pattern of aqueous humor from a POAG patient is shown in Figure 1. Each spot on each gel was defined as the sum of optical densities of each pixel in the spot. After matching and performing a “spot normalization” on the 61 gel images by using user-defined landmarks on the gels, we could perform a statistical analysis (two sample Student *t*-tests) to find the absolute mean spot volumes (the center values, mean 100%, Mean Squared Deviation 100%) that showed a significant difference between glaucoma patients and controls.

### Protein identifications

#### SELDI-TOF biomarker

Protein identification was performed by Ultraflex MALDI-TOF (Bruker, Bremen, Germany) in a similar procedure as described previously [25,26].

For identification, the biomarkers were separated on SDS–PAGE. The band was cut out and cut into two pieces. From one-half the intact protein was eluted for 2 h using an elution buffer containing 50% formic acid, 25% acetonitrile, 15% isopropanol, 10% water, and 0.1% SDS. Afterwards, this sample was re-profiled on SELDI Chips to make sure that the biomarker was present and highly enriched in the elution. The other half was incubated with acetonitrile (100%) followed by centrifugation and subsequent drying in a concentrator (Eppendorf, Hamburg, Germany). Afterwards, they were incubated with trypsin buffer (14.8 µg/µl in 50 mM NH_4_HCO_3_) at 37 °C overnight. The next day, the digested gel pieces were centrifuged, and the supernatants were collected and applied onto an anchor chip target (Bruker, Bremen, Germany) using cinnamic acid matrix (0.02 g cinnamic acid/10 ml in 50% acetonitrile). The MALDI spectra obtained were used for database searches with MASCOT using NCBI (National Institutes of Health, Bethesda, MD) and SwissProt (Swiss Institute of Bioinformatics, Geneva, Switzerland) databases.

#### 2D gel spots

Protein identification was performed as described previously [27,28]. For these identifications, silver staining according to the EMBL (European Molecular Biology Laboratories) protocol was used since this stain is compatible with protein analysis through mass spectrometry [29].

Protein spots of interest were excised from the gels and tryptically digested. The gel pieces were first equilibrated in 25 mM ammonium bicarbonate and then dried in 25 mM ammonium bicarbonate with 50% acetonitrile and 10 mM DTT. Afterwards, the supernatant was removed and 55 mM iodoacetamide was added. The gel pieces were dried, and 12.5 ng/µl trypsin in 25 mM ammonium bicarbonate was added to digest overnight. The resulting peptide fragments were sequenced and identified by electrospray ionization mass spectrometry (ESI-MS/MS; Thermo Electron Corporation, Waltham, MA). All MS/MS spectra were converted into Seaquest files and used for database searches with MASCOT using NCBI and SwissProt databases.

#### Transthyretin detection using ELISA

The existence of transthyretin proteins in aqueous humor of glaucoma patients was confirmed using enzyme linked immunosorbent assay (ELISA; AssayMax Human ProAlbumin ELISA Kit, BioCat GmbH, Heidelberg, Germany). The test was done in accordance with the protocol of the manufacturer, and dilution curves were done to find the right aqueous humor dilution before analysis. Repeat determinations were done for all samples.

## Results

### SELDI-TOF-MS analysis

**Figure 2:**
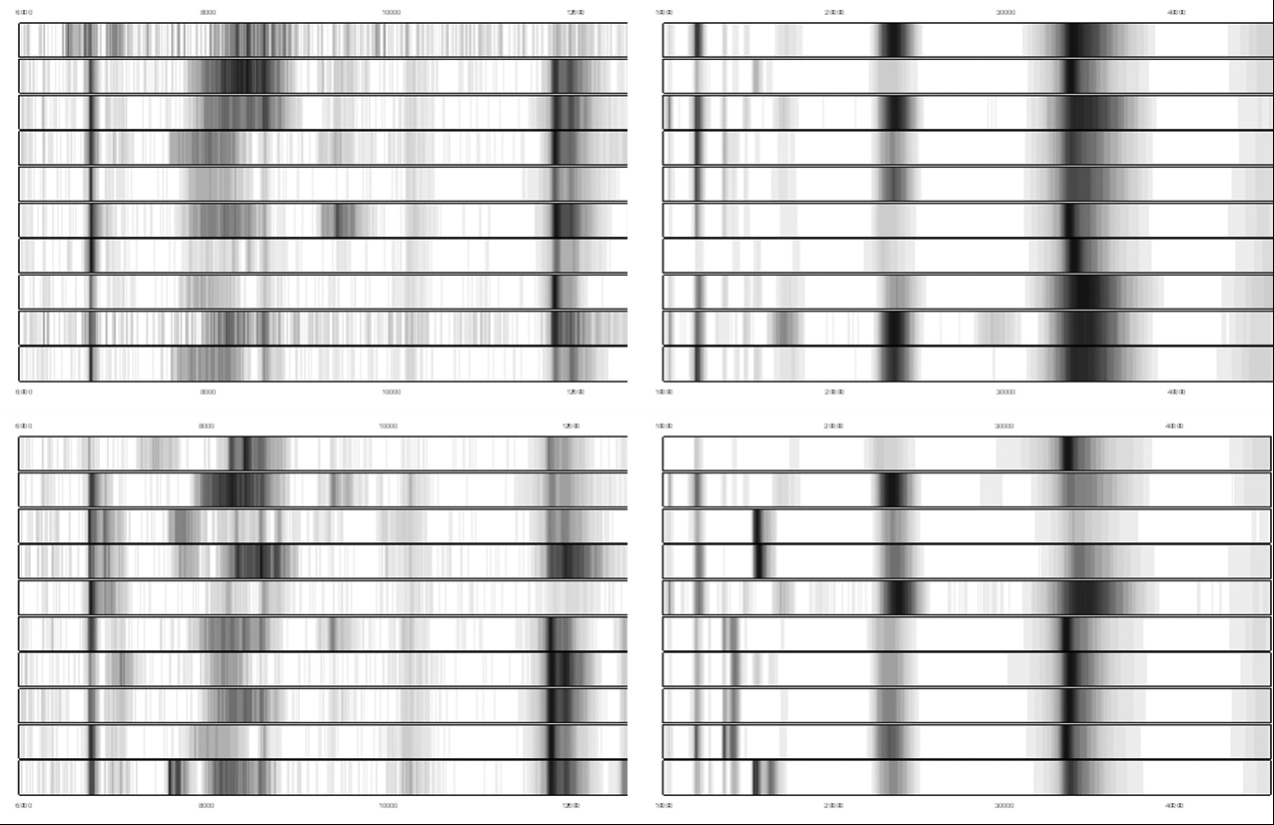
Gel-like views of SELDI-TOF spectra of aqueous humor samples generated on two different chromatographic surfaces. The protein profiles on CM10 (weak cation exchange surface) chips can be seen on the left side and the protein profiles on H50 (reversed phase chemistry) chips on the right side. The profiles on the top are from control subjects and on the bottom from patients with POAG.

**Figure 3:**
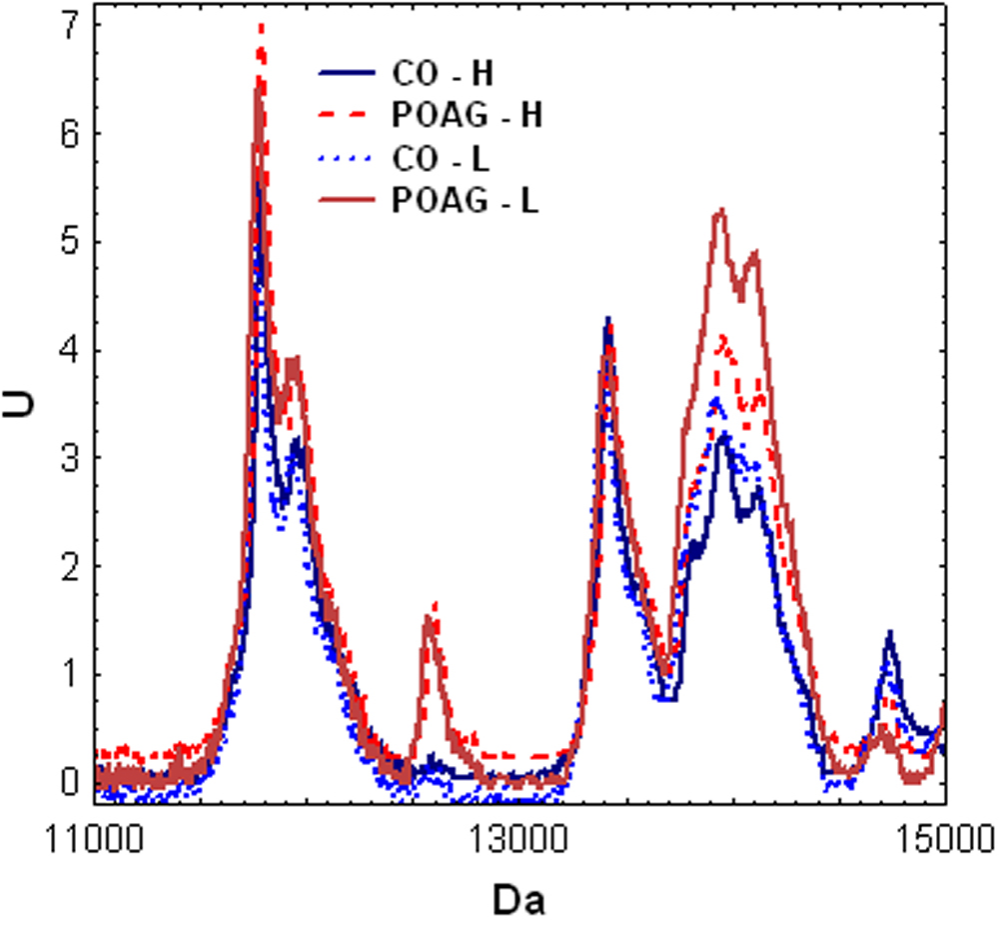
Group average intensities of mass spectrometry measurements for both CO and POAG groups and different laser energies. The intensities of the SELDI-TOF readings (U) were plotted against the molecular weight (in Da). All measurements on CM10 chips are shown in this graph. H: high, L: low energy. In several regions on this graph a higher protein intensity can be seen in the POAG group.

Complex protein patterns could be detected in all samples analyzed with SELDI-TOF-MS. About 250 protein peaks (mass/charge ratios) could be consistently clustered in both groups. Figure 2 shows the gel-like views of aqueous humor samples from POAG patients and controls. Through multivariate statistics, highly significant differences could be detected between the protein profiles of both groups (p=0.000002). The average intensities of the protein profiles for both groups measured on CM10 Chips can be seen in Figure 3. Each Chip was measured with a high (H) and a low (L) intensity protocol.

There were no significant differences between the mean age of the patient groups and the protein pattern of the groups.

**Figure 4:**
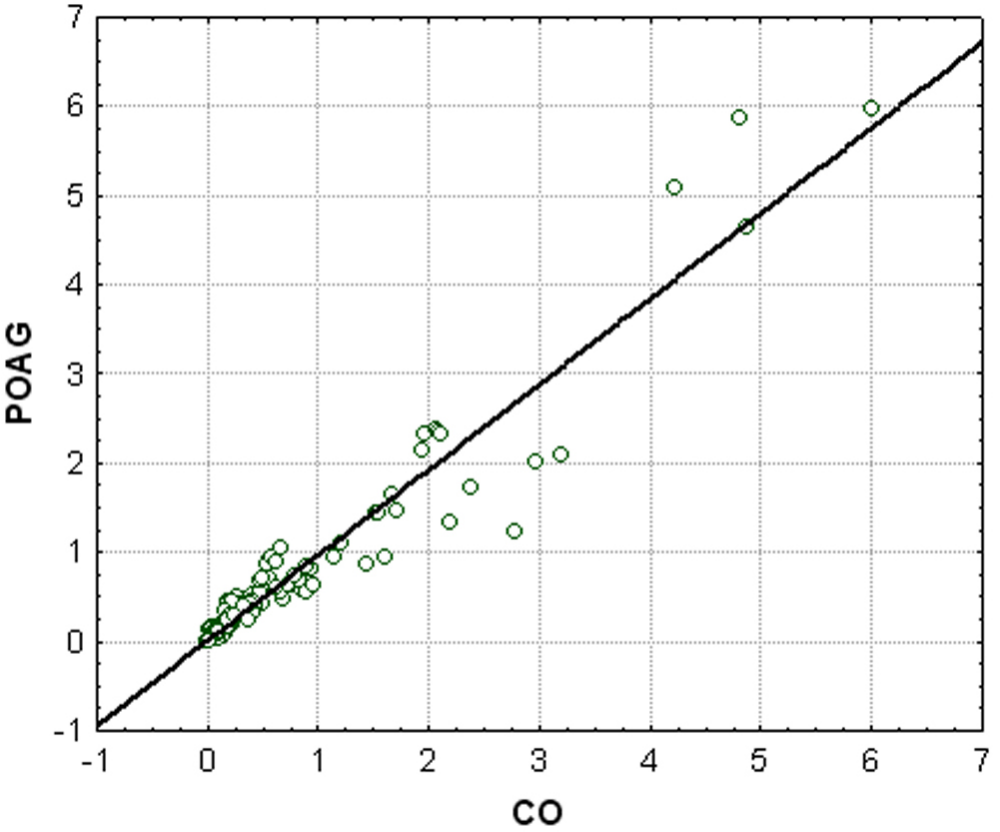
This scatter-plot demonstrates the correlation of the intensities of protein patterns between patients with primary open-angle glaucoma (POAG) and controls (CO). Spots above the line represent protein peaks with higher intensities in the POAG group (using SELDI-TOF analysis), dots below the line are peaks with lower intensity in the POAG group. This graph shows that up- and downregulated proteins were detected in aqueous humor of POAG patients.

The scatter-plot in Figure 4 reveals the correlation between the intensities of peptides and proteins in the POAG and CO groups. All points correspond to a single peptide/protein measured by SELDI-TOF-MS. For each peptide/protein the intensities in the POAG group are plotted versus the intensities in the CO group. All dots below the black line reveal downregulated proteins in POAG, and all points above the line show upregulated proteins in POAG compared to controls.

**Figure 5:**
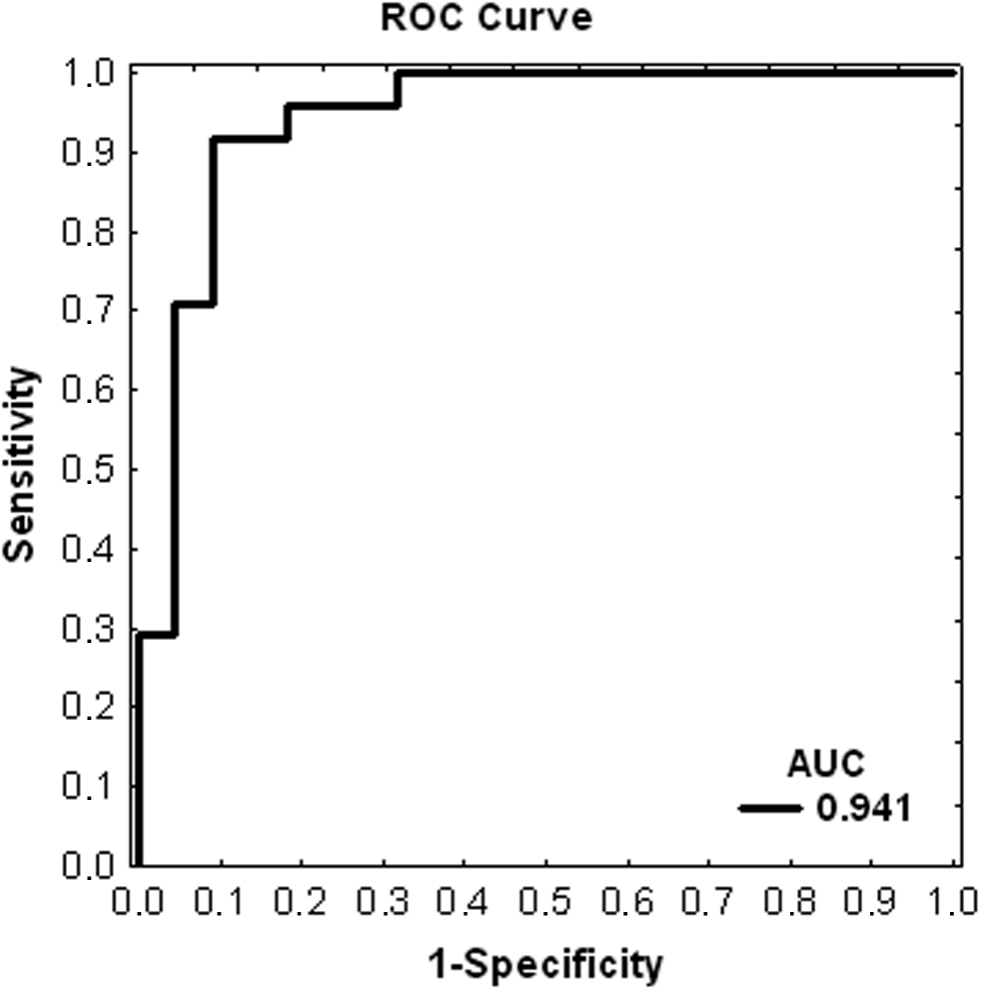
The receiver operating characteristic curve for the diagnosis of glaucoma through complex protein patterns. The groups were discriminated with a sensitivity of 90% and a specificity of 87%. The calculation of the area under the curve (AUC) led to an r value of 0.94. The x-axis shows 1-specificity and the y-axis the sensitivity.

The analyses revealed eight biomarkers that discriminated glaucoma from non-glaucoma controls with a sensitivity of 90% and a specificity of 87%. The area under the curve had an r value of 0.941 (Figure 5).

### 2D gel analysis

We found specific 2D gel patterns with a maximum of 225 protein spots per aqueous humor sample. These patterns revealed significant differences in the proteomic compositions of the aqueous humor from individuals with POAG and without glaucoma (p<0.05).

During electrophoresis, not all compounds of AH were fully resolved. A maximum of 225 spots of polypeptides and proteins could be visualized on the gels in a range from 10-80 kDa and pH 4-7 by using silver staining methods. Funding et al. [15] analyzed AH from patients with corneal rejection by 2D electrophoresis and found a similar protein pattern.

**Figure 6:**
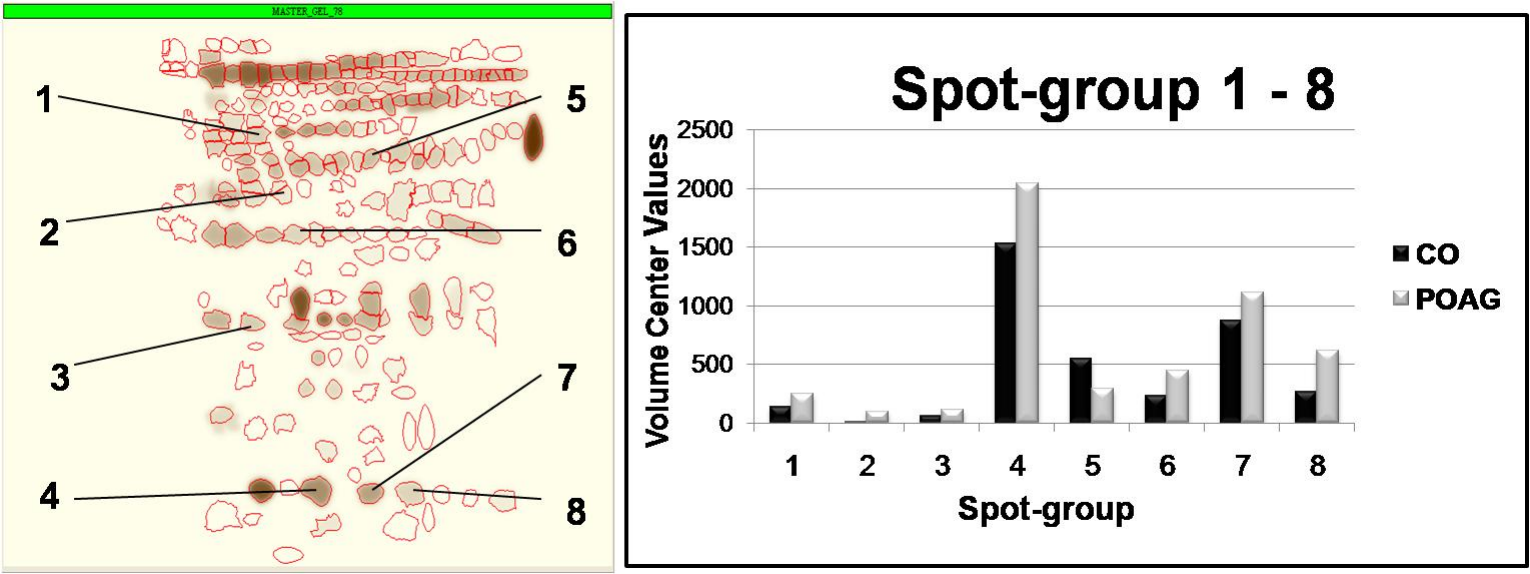
This graph shows the localization of spot groups 1-8 in a “master-gel” on the left side and the mean intensities of the control (CO) and primary open-angle glaucoma (POAG) groups on the right side. The spot groups 4, 7, and 8 existed in all samples and carried a higher amount of protein than all other spot groups especially in the POAG group. The only higher mean volume in controls can be seen in spot group 5.

Each of the 225 spots detected by the 2D gel software was assigned with a unique number to identify spots during the gel matching process afterwards. One hundred and seventy-seven spot groups could be matched. Gels from patients with and without glaucoma contained predominantly the same spots, but in POAG patients, several spots showed a more intense staining. Consequently, there were significant differences in spot volumes compared with those from controls. The differences in mean volume center values of spot groups 1-8 can be seen in Figure 6 as well as their localization in a “master-gel.” Spot group 6 (molecular weight approximately 33 kDa; pH 6) showed one of the highest t-values of 2.3. In this spot group, 51% of the POAG group (mean volume value: 444.2) do not overlap with the CO group (mean volume value: 229.7).

Spot groups 4, 7, and 8 were found in a lower molecular weight range around 16-17 kDa (pH 4.7–5.3). They were detectable in all samples and carried a higher amount of protein than all the other spot groups (Figure 6). The POAG group showed higher mean volume value for these three spot groups especially in spot group 8 where the POAG group showed a mean of 618.9 in comparison to a mean of 263.4 in the control group (t-value=1.5) Only spot group 5 (approximately 45 kDa, pH 5.0) showed an increased mean spot volume for the control group of 545.3 in comparison to a mean volume of 294.9 in the POAG group (t-value=1.4; Figure 6). Fifty-nine percent of the CO group does not overlap with the POAG group.

**Figure 7:**
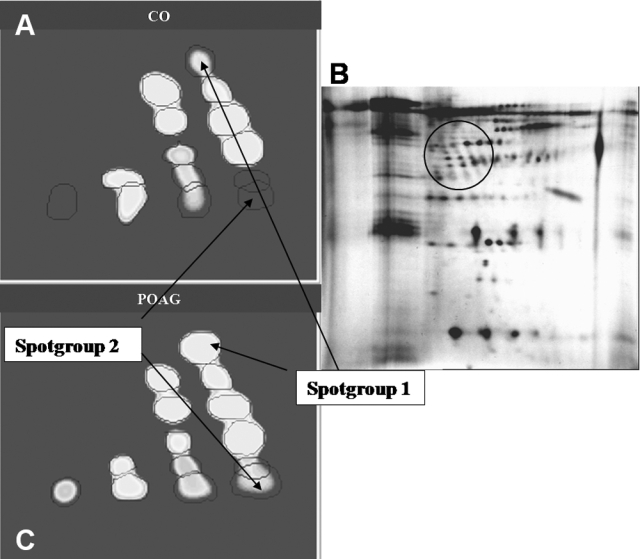
Comparison of spot groups 1 and 2 (molecular weight 37.4–49.4 kDa, IP: 5.7–5.9). **A** shows a synthetic gel of all aqueous humor control samples (CO), and **C** shows a synthetic gel of all aqueous samples from patients with primary open-angle glaucoma (POAG). **B** shows a “real” 2D gel of an aqueous humor sample from a patient with primary open-angle glaucoma. The circle points out spot groups 1 and 2 on this gel. Different staining intensities due to the difference in protein concentration can be seen for spot group 1 and 2 on the two artificial gels.

By creating synthetic gels, we could clearly visualize differences in spot volumes. For example, synthetic gels of spot groups 1 and 2 are shown in Figure 7 (molecular weight area approximately 37-50 kDa; pH 5.7–5.9). While spot group 1 consisted of large, intensive spots in individual glaucoma gels carrying a high amount of protein, spots from spot group 2 were smaller, less intensive, and not to be found on all gels. Therefore, spot group 1 is shown with a big radius in the synthetic gel of the POAG group (Figure 7C). Forty-four percent of POAG is not overlapping with the CO group (t-value=1.4). Spot group 2 in contrast is much smaller; here, 77% of POAG were not overlapping with controls.

### Identifications

**Figure 8:**
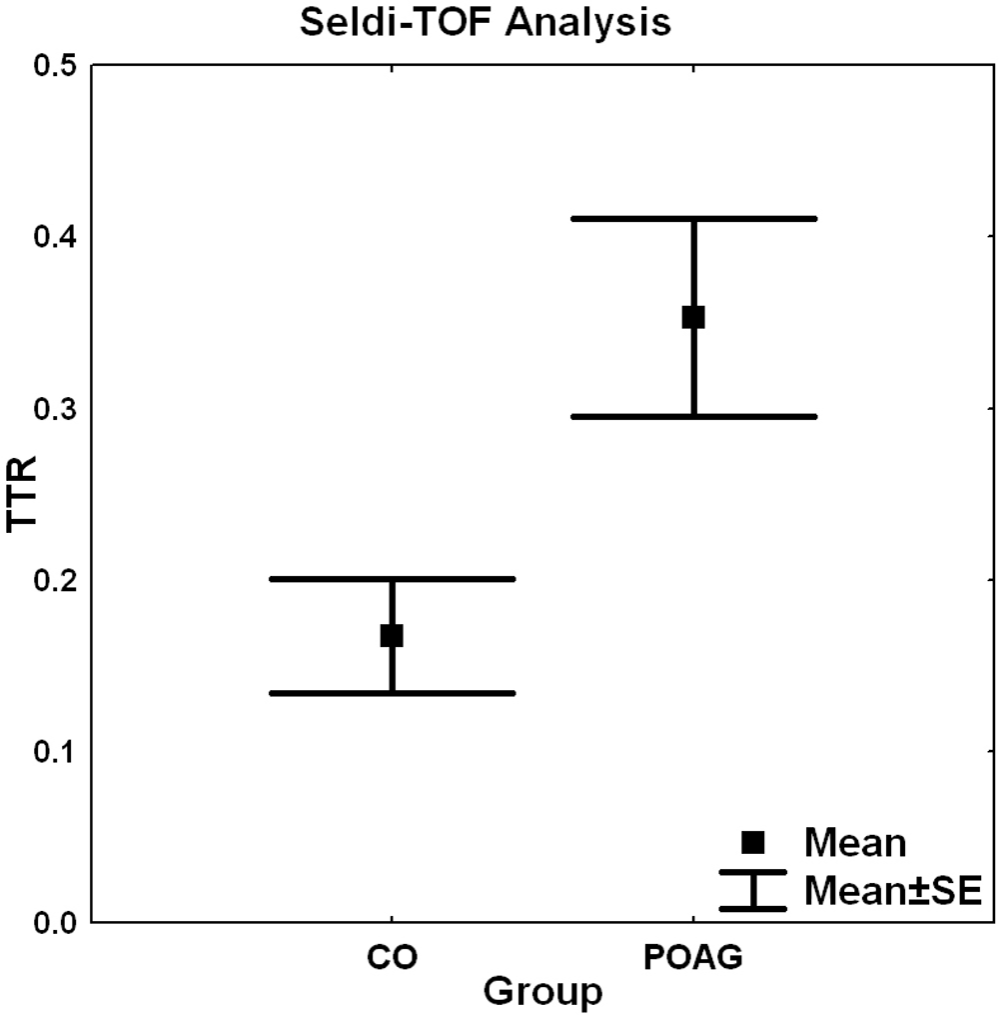
Box plot of the protein concentration of the aqueous humor samples (mean±SE) of the control group and the primary open-angle glaucoma group at 14,132 Da from SELDI-TOF analysis. This protein was significantly upregulated in the POAG group (p=0.006) and was identified as human transthyretin (TTR) through MALDI-TOF.

The SELDI-TOF-MS analysis of the aqueous humor protein profiles revealed a significant upregulated protein concentration at 14,132 Da in the POAG group in comparison to controls (p=0.006; Figure 8). This protein peak was detected as a biomarker through multivariate statistics and was later significantly identified as transthyretin (TTR) by mass spectrometry (MALDI-TOF, Mascot Score=250).

Spot groups 4 and 7 in the 2D gel analysis showed a higher protein concentration in aqueous humor samples from patients with POAG (Figure 6). The protein in these spots was significantly identified as TTR by mass spectrometry (ESI-MS/MS) with a Mascot Score of 205 and with seven peptides sequenced.

### ELISA

**Figure 9:**
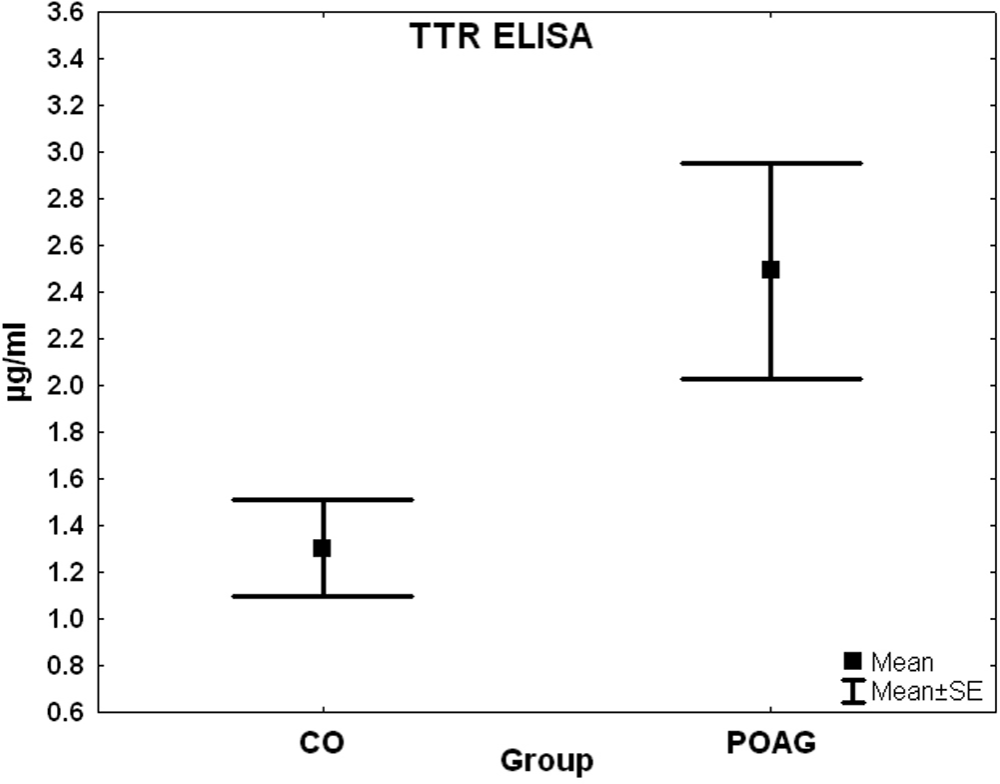
Box plot of transthyretin concentration measured by ELISA. Levels of transthyretin protein (mean±SE) were plotted for control subjects (CO) and patients with primary open-angle glaucoma (POAG). The protein was detected using a specific ELISA. The POAG group showed significantly higher levels of TTR then the control group (p=0.03).

The CO group showed a mean of 1.3 µg/ml TTR protein and the POAG group a mean of 2.5 µg/ml TTR protein detected through ELISA. The POAG group showed significantly higher levels of TTR than the control group (p=0.03; Figure 9).

## Discussion

Since the aqueous humor is located closer than serum to the site of damage in glaucomatous eyes, its analysis might give us further information about the pathogenesis of the disease. The quantity of proteins in the aqueous humor is very limited; therefore, very sensitive analytical methods such as SELDI-TOF-MS and 2D gel electrophoresis were used to compare proteins patterns of POAG patients and controls.

Other studies also used 2D electrophoresis for aqueous humor analysis. Funding et al. [15] analyzed the proteins in the aqueous humor of patients with corneal rejection with 2D electrophoresis. They found a significant difference in protein composition in patients in comparison to control subjects, some of the increased protein spots derived from albumin, cytokeratin type II, and alpha1-antitrypsin. In another study, 2D electrophoresis was also used to analyze AH humor samples, and in this case, it was in patients with myopia [18]. One of the later identified proteins using ESI-MS/MS, was TTR, which was significantly increased in patients with myopia. We also used 2D electrophoresis to analyze AH humor samples of patients with POAG. Protein separation through 2D electrophoresis revealed complex protein patterns in both groups (POAG and CO). One hundred and seventy-seven spot groups could be matched. In eight spot groups, we found significant differences of mean spot volumes between glaucoma patients and controls. For several spot groups such as group 6 (approximately 33 kDa) or group 7 and 8 (both 16–17 kDa), the POAG group showed a higher mean volume value than the CO group (Figure 6). The mean volume value of the glaucoma group was lower than that of the control group only in spot group 5 (approximately 45 kDa; Figure 6).

Using SELDI-TOF-MS methods, we detected a highly significant difference between protein profiles of the POAG group and the CO group (p=0.000002). Eight biomarkers were calculated with multivariate statistical methods that discriminated glaucoma from non-glaucoma controls with a sensitivity of 90% and a specificity of 87% (Figure 5).

In both studies, TTR was one of the significantly upregulated biomarkers in the POAG group in comparison to the control group (p=0.006). This marker was identified with two different methods, MALDI-TOF-MS and ESI-MS/MS. These results were also confirmed using ELISA, which detected a significantly higher level of TTR in the POAG group (p=0.039; Figure 9).

TTR is a tetrameric plasma protein and usually responsible for the transportation of thyroxine and retinol [30]. Many pathologic and non-pathologic TTR variations are already described including TTR variations with specific gene mutations that are associated with familial amyloidosis [31]. Some of these variations were found by using IEF screening [32]. Sometimes TTR and its variants lead to extracellular polymerization of insoluble protein fibrils called amyloid deposits. The mechanism of the accumulation is still not known [33-35]. Our findings of increased levels of TTR in the aqueous humor of glaucoma patients indicate that this protein might play a role in the pathogenesis of glaucoma. Interfering protein precipitations of TTR might cause a mechanical barrier for aqueous fluid that could consequently lead to glaucoma in some patients.

There are some theoretical explanations about elevated TTR levels in glaucomatous AH. In animal experiments, TTR synthesis in ciliary pigment epithelium could be shown [36]. This location of TTR synthesis could explain the local influence of this protein in the anterior chamber of the eye without having a systemic effect. Another study on rats showed a widespread ocular distribution of TTR including retinal pigment epithelium and retinal ganglion cells [37]. Possibly dying retinal ganglion cells in glaucoma patients dispense their TTR in the AH, which leads to higher TTR concentrations in patients. Furthermore, in an animal study, TTR genes were shown to be expressed more in diabetic Müller cells when compared to non-diabetic rats [38].

Transthyretin could be identified in several studies. It was identified as a plasma biomarker in a SELDI-TOF study in patients with rheumatoid arthritis [39]. Increased levels of TTR have been detected in cerebrospinal fluid in a protein complex consisting of prostaglandin-d-synthase and transthyretin. In Alzheimer patients increased levels of TTR have been detected in cerebrospinal fluid in a protein complex consisting of prostaglandin-d-synthase and transthyretin or by itself [40,41]. Other studies report lower levels of TTR in cerebrospinal fluid of Alzheimer patients in comparison to controls [42]. Leptomeningeal metastasis of breast cancer patients showed decreased levels of TTR [43], and a 2D electrophoresis study also showed decreased levels of TTR in cerebrospinal fluid of Guillain-Barré syndrome patients [44].

The findings of this study suggest that there are significant differences in the concentration of TTR in the aqueous humor between POAG patients and controls. These results lead to the assumption that there is a connection between elevated concentrations of TTR and the pathogenesis of POAG.
